# Absolute vs. Weight-Related Maximum Oxygen Uptake in Firefighters: Fitness Evaluation with and without Protective Clothing and Self-Contained Breathing Apparatus among Age Group

**DOI:** 10.1371/journal.pone.0119757

**Published:** 2015-03-12

**Authors:** Fabrizio Perroni, Laura Guidetti, Lamberto Cignitti, Carlo Baldari

**Affiliations:** 1 School of Exercise and Sport Sciences (SUISM), Department of Medical Sciences, University of Turin, Turin, Italy; 2 Department of Movement, Human and Health Sciences, University of Rome “Foro Italico”, Rome, Italy; 3 Italian Fire Fighter Corp, Rome, Italy; New York University School of Medicine, UNITED STATES

## Abstract

During fire emergencies, firefighters wear personal protective devices (PC) and a self-contained breathing apparatus (S.C.B.A.) to be protected from injuries. The purpose of this study was to investigate the differences of aerobic level in 197 firefighters (age: 34±7 yr; BMI: 24.4±2.3 kg.m^-2^), evaluated by a Queen’s College Step field Test (QCST), performed with and without fire protective garments, and to analyze the differences among age groups (<25 yr; 26-30 yr, 31-35 yr, 36-40 yr and >40 yr). Variance analysis was applied to assess differences (p < 0.05) between tests and age groups observed in absolute and weight-related values, while a correlation was examined between QCST with and without PC+S.C.B.A. The results have shown that a 13% of firefighters failed to complete the test with PC+S.C.B.A. and significant differences between QCST performed with and without PC+S.C.B.A. in absolute (F_(1,169)_ = 42.6, p < 0.0001) and weight-related (F_(1,169)_ = 339.9, p < 0.0001) terms. A better correlation has been found in L•min^-1^ (r=0.67) than in ml•kg^-1^•min^-1^ (r=0.54). Moreover, we found significant differences among age groups both in absolute and weight-related values. The assessment of maximum oxygen uptake of firefighters in absolute term can be a useful tool to evaluate the firefighters' cardiovascular strain.

## Introduction

Firefighting is one of the most hazardous civilian occupations, implying variable working conditions under time urgency. Several studies [1,2, 3, 4, 5, 6] have shown that the combination of physical activity and/or exposure to external heat sources causes an increased physiological and psychological stress.

During fire emergencies, firefighters wear personal protective devices (PC), composed by a layered thermal protective clothing (flame resistant outer shell and insulating thermal liner), a heavy footwear, a helmet, and a self-contained breathing apparatus (S.C.B.A.). Despite PC+SCBA are a good barrier for the firefighters from thermal radiation, burns, injuries, smoke and noxious gases these can have negative effects on gait, metabolic and thermal efficiency, fatigue leading to a significant reduction in work capability and work duration [7, 8, 9, 10, 11, 12, 13].

Among the different parameters the maximum oxygen uptake (VO_2max_) is the most frequent variable taken into account. Cardiovascular strain has been discussed in response to a real and simulated emergency and during different tasks [7, 8, 14, 15, 16]. In particular, single firefighters’ activities with PC+ SCBA showed an average VO_2_ of 39.0 ml^.^kg^-1.^min^-1^ while climbing stairs, a VO_2_ of 23.4–25.7 ml^.^kg^-1.^min^-1^ lifting and moving the hose, a VO_2_ of 30.9 ml^.^kg^-1.^min^-1^ controlling a flexible tube and a VO_2_ of 36.6–44.0 ml^.^kg^-1.^min^-1^ transporting equipment on the stairs climb [1, 17]. Perroni et al. [8], analyzing simulated emergencies of Italian firefighters, have shown that the values of VO_2_ remained elevated after 30 min of rest (VO_2_: 8.86 ± 2.67 ml^.^kg^-1.^min^-1^) compared with basal values (VO_2_: 4.57 ± 1.07 ml^.^kg^-1.^min^-1^). Furthermore, it has been shown in firefighters a constant and annual physiological decrease related to age [18, 19, 20]. For these reasons, numerous studies [21, 22, 23, 24, 25] have suggested VO_2max_> 33 ml^.^kg^-1.^min^-1^, with preferably >45 ml^.^kg^-1.^min^-1^, to successfully complete rescue activities.

Although the most accurate method to determine the aerobic power of individuals is the direct measurement of VO_2max_, some aspects may limit the applicability of large-scale assessments such as a lot of time to carry out the test, sophisticated and expensive equipment, and large effort of individuals for the attainment of VO_2max_. In contrast, the field trials are inexpensive, easy to administer and might be best suited to examine large number of firefighters in minimum time.

Several studies [23, 26, 27, 28, 29] have demonstrated that evaluation of VO_2max_ in weight-related rate (ml kg^-1^ min^-1^) is fundamental for job performance. Given that job performance could be influenced by body weight, by weigh of PC+SCBA and/or by further weight of supplementary fire device (i.e., operating high pressure hoses, ladders, lifting weights), the purposes of this study were; 1) analyze the utility of evaluate the maximum oxygen uptake of Italian firefighters recruits in absolute or weight-related terms by a Queen’s College Step field Test (QCST) (performed with and without wearing fire protective garments), 2) compare the effects of wearing fire protective garments and 3) analyze the differences among age groups.

## Methods

### 2.1 Participants

One hundred and nineteen-seven male Italian firefighters recruits during the residential Italian Fire Fighter Corp training course, decided to be evaluated. The subjects had the following general baseline characteristics (mean ± SD): age 34±7 yr, height 177±6 cm, weight 76.1±8.6 kg, BMI 24.4±2.3 kg^.^m^-2^. All subjects were divided into five different age groups, under 25-year-old (<25 yr), 26- to 30-year-old (26–30 yr), 31- to 35-year-old (31–35 yr), 36- to 40-year-old (36–40 yr) and over 40 year-old (>40 yr).

### 2.2 Procedures

The study was approved by the scientific Institutional Review Board of United Hospitals, University Hospital of Foggia with the goal to investigate differences in the aerobic firefighters' evaluation analyzing the differences among age groups.

Fitness evaluations were administered in two experimental sessions with a gap of a week in the middle of the residential Italian Fire Fighter Corp training course (i.e. May-June). The first session included anthropometric (i.e., weight, height and body mass) evaluation and the aerobic power test performed without PC + S.C.B.A (sneakers, socks, shorts and cotton t-shirt), while the second session aimed to evaluate the aerobic power with European Fire Protection Agency standard PC + S.C.B.A (EN 531 A, B1, C1, EN 469/97, EN 469/95). Beneath the PC, the firefighters wore underwear, socks, standard issue cotton station long pants, and a cotton t-shirt. The total weight of the ensemble was approximately 23 kg.

Given that various studies have shown that maximal heart rate declines with increasing age, we used age-correction factor of Åstrand [30] to compare the results of the aerobic power of the different age groups estimated by QCST.

All participants were adequately informed about the study and gave their written informed consent and answered to an exercise/medical history questionnaire (i.e. activity level, educational background, dietary habits, tobacco smoking and alcohol consumption, and medication and history of physical activity). Prior to the evaluation, firefighters underwent a standardized warm-up period lasting 15-minutes, composed of low intensity running followed by strolling locomotion and stretching of the lower limb muscles. All the experimental tests were performed in the morning (between 9.30 and 11.00am) at 22–24°C and 50–60% relative humidity to eliminate circadian rhythms, nutrition and climate-related factors.

#### 2.2.1 Anthropometric evaluations

Weight and height were measured in light clothes, barefoot using an electronic scale to the nearest 0.1 kg and a fixed stadiometer to the nearest 0.1 cm. It was used the Body Mass Index (BMI) to measure weight relative to height and it was calculated dividing the body mass by height in squared meters (kg/m^2^).

#### 2.2.2 Aerobic Evaluation

The VO_2max_ of firefighters was assessed by the QCST [31] that showed a significant correlation (r = 0.95) with VO_2max_ directly measured on bicycle ergometer [32].

The firefighters were required to perform 3 min of step up and down on a step of 40 cm at a frequency of 24 steps^.^min^-1^. At the end of the test, heart rate was recorded from 5 to 20 s of the recovery phase (HR_post exercise_). It was used the following formula to estimate VO_2max_:

VO2max(ml.kg−1.min−1) = 111.30− (0.42*HRpost exercise).

Then, to obtain the VO_2max_ in L^.^min^-1^ we have calculated:

VO2max(L.min−1) = (VO2max (ml.kg−1.min−1)*body weight (kg))/1000

In this test a lower heart rate after exercise corresponds to a higher estimate VO_2max_.

### 2.3 Statistical Analysis

Descriptive statistics (means, standard deviations and ranges) were computed to provide the physical fitness profile for each measured parameter. Since the data showed a normal distribution were implemented parametric tests. Throughout the study were selected 0.05 confidence levels.

Analysis of Variance (ANOVA) was applied to assess differences between tests (with and without PC+S.C.B.A.) and between age group (<25 yr, 26–30 yr, 31–35 yr, 36–40 yr and >40 yr) observed in absolute and weight-related values. Cohen’s effect sizes (ES) were calculated to provide meaningful analysis for comparisons among groups. Values ES ≤0.2, from 0.3 to 0.6, <1.2 and >1.2 were considered trivial, small, moderate and large [33].

A correlation coefficients (r) was examined between QCST performed by all firefighters with and without PC+S.C.B.A. and interpreted in accordance with the following scale of magnitude [34]: trivial (r ≤ 0.1), small (0.1 ≤ r < 0.3), moderate (0.3 ≤ r < 0.5), large (0.5 ≤ r < 0.7), very large (0.7 ≤ r < 0.9), nearly perfect (r ≥ 0.9) and perfect (r = 1).

## Results

Means, standard deviations and statistical differences (p<0.05) of anthropometric and aerobic profile of the participant to the study are shown in Table 1.

**Table 1:** Means ± standard deviations of anthropometric and aerobic data across age group.

	*Age group (yr)*					*total*
*Variables*	*<25*	*26–30*	*31–35*	*36–40*	*>40*	
	*n = 28*	*n = 32*	*n = 46*	*n = 32*	*n = 32*	*n = 170*
	*range*: *22–25*	*range*: *26–30*	*range*: *31–35*	*range*: *36–40*	*range*: *41–47*	*range*: *22–47*
	Mean ± SD	Mean ± SD	Mean ± SD	Mean ± SD	Mean ± SD	Mean ± SD
***Weight (kg)***	74.3 ± 7.9	73.8 ± 6.1	74.9 ± 8.2	77.9 ± 8.7	77.8 ± 9.9	75.6 ± 8.4
***High (cm)***	178 ± 5	176 ± 6	176 ± 6	178 ± 5	177 ± 6	176 ± 1
***BMI (kg.m-2)***	23.4 ± 2.0	23.7 ± 1.6	24.2 ± 2.3	24.7 ± 2.3	24.8 ± 2.6	24.2 ± 2.2
***Queen's (ml.kg-1.min-1)***	53.3 ± 5.9* ^§^ ^#^	51.8 ± 8.5^§^ ^#^	48.9 ± 6.5^#^	46.1 ± 5.9	43.7 ± 4.7	48.7 ± 7.1
***Queen's (L.min-1)***	3.9 ± 0.6* ^§^ ^#^	3.8 ± 0.7^#^	3.7 ± 0.6	3.6 ± 0.6	3.4 ± 0.6	3.7 ± 0.6
***Queen's S.C.B.A. (ml.kg-1.min-1)***	42.3 ± 3.9* ^§^ ^#^	42.7 ± 4.8* ^§^ ^#^	39.4 ± 4.4^#^	38.1 ± 4.3#	35.8 ± 5.4	39.9 ± 5.4
***Queen's S.C.B.A. (L.min-1)***	4.3 ± 0.5* ^§^ ^#^	4.2 ± 0.6* ^§^ ^#^	3.8 ± 0.5	3.8 ± 0.6	3.6 ± 0.6	3.9 ± 0.6

* = *p*≤*0*.*05 V*_*S*_ *31–35*

^§^ = *p*≤*0*.*05 V*_*S*_ *36–40*

^#^ = *p*≤*0*.*05 V*_*S*_ *>40*

Wearing the PC+S.C.B.A., 26 (13%) of all firefighters failed to complete the test and have been excluded from the total data analysis (Table 2).

**Table 2:** Firefighters (n°), number (n° failed) and percentage (%) of Firefighters who did not complete the Queen’s College step test with PC+ S.C.B.A.

*Age group (yr)*	*Firefighters*			
	*n°*	*n° failed*	*% of age group*	*% of total*
***<25***	30	2	7	1
***26–30***	35	3	9	2
***31–35***	53	7	13	4
***36–40***	38	6	16	3
***>40***	40	8	20	4
***Total***	***196***	***26***		**13**

Comparing the average values of weight-related VO_2max_ of tests without (VO_2max_ = 48.7 ± 7.1 ml^.^kg^-1.^min^-1^) and with PC+S.C.B.A. (VO_2max_ = 39.9 ± 5.4 ml^.^kg^-1.^min^-1^) of all firefighters, we can notice a sharp decrease (22%) between the two. In contrast, the average values of absolute VO_2max_ evaluated without (VO_2max_ = 3.7 ± 0.6 L^.^min^-1^) and with PC+S.C.B.A. (VO_2max_ = 3.9 ± 0.6 L^.^min^-1^), we can notice an increase of 6% between test.

In particular, the higher percentage differences were observed between 26–30 yr vs 31–35 yr and between 36–40 yr vs >40 in test performed with PC+S.C.B.A. both in absolute (9.5% and 5.3%, respectively) and weight related (7.7% and 6.0%, respectively) terms. Test performed without PC+S.C.B.A. have shown the higher percentage differences between 26–30 yr vs 31–35 yr and between 36–40 yr vs >40 in absolute term (5.2% and 5.5%, respectively), whereas between 26–30 yr vs 31–35 yr and between 31–35 yr vs 36–40 in weight related values (5.9% and 5.7%, respectively).

VO_2max_ values showed significant differences between QCST performed with and without PC+S.C.B.A. in absolute (F_*(1*,*169)*_ = 42.6, p < 0.0001, ES = 0.19) and weight-related (F_*(1*,*169)*_ = 339.9, p < 0.0001, ES = 0.57) terms.

Analysing all subjects, the tests performed with and without PC+S.C.B.A. have shown a better correlation coefficient in L min^-1^ (r = 0.67) than in ml kg^-1^ min^-1^ (r = 0.54). Across age groups, correlation between tests performed with and without PC+S.C.B.A. observed in absolute and weight-related values showed the same trend: <25 yr (r = 0.66 and r = 0.39, respectively), 26–30 yr (r = 0.71 and r = 0.54, respectively), 31–35 yr (r = 0.61 and r = 0.43, respectively), 36–40 yr (r = 0.60 and r = 0.25, respectively), and >40 yr (r = 0.58 and r = 0.25).

## Discussion

The purpose of this preliminary study was to evaluate the maximum oxygen uptake (absolute and weight-related) of Italian firefighters recruits performed with and without wearing PC+S.C.B.A. and to analyze the differences among age groups. The main findings of this study were the statistical differences between tests performed with and without PC+S.C.B.A. in Italian firefighters recruits and a high correlation between test observed in absolute value.

It is generally recognized that VO_2max_ is the single best physiological indicator of muscular capacity for sustained work and that the assessment of VO_2max_ can determine workers’ cardiovascular health and physical capability required to carry out their duties safely and effectively. Consequently different exercise protocols are used to determine VO_2max_ for assessment of cardiovascular fitness, prescribed training programs and evaluate his effects on the health in an occupational setting.

Since the most precise method of measuring VO_2max_ is a maximal laboratory exercise test and it isn’t always possible to do, we used the QCST as it is recommended as a valid method to evaluate cardiorespiratory fitness for large numbers of population [30, 35] and the movement was similar to that used for the job performance of the firefighters (i.e. climbing stairs).

Even if we have found an increased number of firefighters who failed to complete the test with PC+S.C.B.A., they showed the lowest values compared with Perroni et al. [36, 37]. Percentage differences between the values recorded with and without PC+S.C.B.A. and observed in weight-related values, have shown a higher decrease in 31–35 yr (19%) compared with the other categories (<25 yr = 17%; 26–30 yr = 17%, 36–40 yr = 17% and >40 yr = 18%). In contrast, differences between tests observed in absolute terms have demonstrated a higher increase of values in <25 and 26–30 yr (10% and 11%, respectively) compared with 31–35 yr (3%), 36–40 yr (6%) and >40 yr (6%). Punakallio et al. [38] have found that the average annual change aerobic capacity, in male Finnish firefighters (30–44 yrs) at 3- and 13-year follow-ups, was −1.12% in absolute (L^.^min^−1^) and −1.33%. in weight-related (ml^.^kg^−1.^min^−1^). A previous study by Perroni et al. [37] had hypothesized that decreased values of performance and increased percentage of failure were due to premature muscle fatigue of the lower limbs muscle, caused by an overload of the musculoskeletal system and thermoregulation, rather than by a deficit of cardiovascular system. As fatigued muscles can affect lifting techniques and can increase the risk of injury [39], this is a parameter that has to be taken into account while training programs. In this direction, Griefahn et al. [40] declare that at the same extra-load the subjects with elevated body mass (i.e. height and weight) are less affected by the load carried with lower cardiovascular strain.

In this study, the aerobic power of all firefighters measured without PC+S.C.B.A. showed lower absolute (3.7 Vs 4.6 L^.^min^-1^) and weight-related (48.7 Vs 58 ml^.^kg^-1.^min^-1^) values than the values investigated by Lindberg et al. [41] on full-time firefighters (mean age: 34 yrs, range: 20–57) by submaximal treadmill running test. In addition, in the same study Lindberg et al. [41] found a correlation between work tasks and absolute and weight-related VO_2max_.

Wearing PC+S.C.B.A., all ranges of age of firefighters were still within the minimum values, but below the preferable values recommended for the successful completion of a standard rescue protocol. Otherwise, absolute VO_2max_ data have shown values in line (2.7–4.0 L^.^min^-1^) with those proposed for firefighters by O’Connell et al. [17] and von Heimburg et al. [26].

Results of all our firefighters were lower than Perroni et al. [36, 37] and, analyzing the aerobic values of each age category, than Perroni et al. [37] for 31–35 yrs, 36–40 yrs and >40 yrs. The firefighters values examined in this investigation are substantially higher than the values measured by Swank et al. [42] on a cycle ergometer and by Hammer and Heath [43] on a treadmill for the same mean age.

Given that various studies [21, 44, 45, 46, 47, 48] have shown a correlation between high levels of fitness and improved job performance during actual firefighting activities and a decreased risk of injury, a periodic fitness evaluation of firefighters should be done. Although the aim of the tests is to measure specific physical capacities and evaluate the relation between different tests, a high number of experiments could be time consuming and may be physically and mentally strenuous for the firefighters. A reduction in tests number and the choice of an adequate test could be useful for the organization of specific and periodic assessment of physical fitness of firefighters. Given that 1) in this study we have found a large correlation coefficient between tests performed with and without PC+ S.C.B.A observed in L min^-1^ (r = 0.67), 2) Perroni et al. [8] found the same values as in our test performed with PC+ S.C.B.A. observed in absolute rate and 3) the absolute VO_2max_ does not take into account the body weight of subjects and the heavy PC+S.C.B.A., we suggest that the QCST performed with PC+ S.C.B.A. observed in absolute term can be an useful tool to evaluate the cardiovascular strain of the firefighters.

## Conclusion

Our results show that there are significant differences among step tests performed with and without PC+S.C.B.A., observed in absolute and weight-related rate, and that a simple procedure can be carried out for the evaluation of VO_2 max_ in large number of firefighters. A systematic and periodic assessment of fitness level of firefighters with PC+S.C.B.A. (by a correlate field test) could allow us to create specific training programs and monitor the evolution of the physical capabilities of firefighters during the job career to perform their occupational activities safely reducing the risk of injuries. Moreover, the evaluation of VO_2max_ observed in L^.^min^-1^ could facilitate a comparison between the same firefighters and different subjects during a specific period of physical conditioning.
